# State-Dependent Effects of Ventromedial Prefrontal Cortex Continuous Thetaburst Stimulation on Cocaine Cue Reactivity in Chronic Cocaine Users

**DOI:** 10.3389/fpsyt.2019.00317

**Published:** 2019-05-08

**Authors:** Tonisha E. Kearney-Ramos, Logan T. Dowdle, Oliver J. Mithoefer, William Devries, Mark S. George, Colleen A. Hanlon

**Affiliations:** ^1^Division on Substance Use Disorders, Columbia University Irving Medical Center, New York, NY, United States; ^2^Department of Psychiatry, Medical University of South Carolina, Charleston, SC, United States; ^3^Department of Neurosciences, Medical University of South Carolina, Charleston, SC, United States; ^4^Center for Biomedical Imaging, Medical University of South Carolina, Charleston, SC, United States; ^5^Ralph S. Johnson VA Medical Center, Charleston, SC, United States

**Keywords:** addiction, functional magnetic resonance imaging, repetitive transcranial magnetic stimulation, inhibitory, neural circuit, independent component analysis

## Abstract

Cue-induced craving is a significant barrier to obtaining abstinence from cocaine. Neuroimaging research has shown that cocaine cue exposure evokes elevated activity in a network of frontal-striatal brain regions involved in drug craving and drug seeking. Prior research from our laboratory has demonstrated that when targeted at the medial prefrontal cortex (mPFC), continuous theta burst stimulation (cTBS), an inhibitory form of non-invasive brain stimulation, can decrease drug cue-related activity in the striatum in cocaine users and alcohol users. However, it is known that there are individual differences in response to repetitive transcranial magnetic stimulation (rTMS), with some individuals being responders and others non-responders. There is some evidence that state-dependent effects influence response to rTMS, with baseline neural state predicting rTMS treatment outcomes. In this single-blind, active sham-controlled crossover study, we assess the striatum as a biomarker of treatment response by determining if baseline drug cue reactivity in the striatum influences striatal response to mPFC cTBS. The brain response to cocaine cues was measured in 19 cocaine-dependent individuals immediately before and after real and sham cTBS (110% resting motor threshold, 3600 total pulses). Group independent component analysis (ICA) revealed a prominent striatum network comprised of bilateral caudate, putamen, and nucleus accumbens, which was modulated by the cocaine cue reactivity task. Baseline drug cue reactivity in this striatal network was inversely related to change in striatum reactivity after real (vs. sham) cTBS treatment (ρ = -.79; *p* < .001; *R* ^2^_Adj_ = .58). Specifically, individuals with a *high* striatal response to cocaine cues at baseline had significantly *attenuated* striatal activity after real but not sham cTBS (*t*_9_ = -3.76; *p* ≤ .005). These data demonstrate that the effects of mPFC cTBS on the neural circuitry of craving are not uniform and may depend on an individual’s baseline frontal-striatal reactivity to cues. This underscores the importance of assessing individual variability as we develop brain stimulation treatments for addiction.

## Introduction

Substance dependence is a chronic, relapsing brain disease characterized by compulsive drug seeking and use behaviors, despite harmful consequences (1). Cocaine use disorder (CUD) is among the most difficult substance use disorders to treat. The lack of FDA-approved pharmacotherapies, and limited efficacy of conventional psychotherapies, means that as many as 70% of treatment-seeking cocaine users relapse within the first 3 months (1). This leaves cocaine-dependent individuals with limited support for overcoming their chronic illness. Consequently, there is a pressing need for innovative treatment development, including approaches that specifically target the neural circuits associated with continued, habitual use in this population.

One of the strongest precipitants of relapse is drug cue-induced craving (1–4). Craving is associated with activity in reward-motivation brain regions, including the medial prefrontal cortex (mPFC) and striatum (1, 5, 6). Chronic cocaine users exhibit elevated activity in reward-motivation circuitry when exposed to drug-related cues (1, 5, 7). Functional neuroimaging studies have shown that the level of activity in this circuit is related to the intensity of craving (8, 9),and can reliably predict relapse in treatment-seeking substance users (1, 4, 10). Thus, one way to effectively treat CUD may be through a more targeted neurobiological approach, such as by directly modulating activity in this mPFC-striatal craving circuit.

Repetitive transcranial magnetic stimulation (rTMS) is a non-invasive brain stimulation technique that can be used to selectively modulate cortical and subcortical brain activity. Theta burst stimulation (TBS) is a patterned variant of rTMS that mimics endogenous neuronal firing patterns associated with learning and memory (11, 12). Depending on the TBS delivery pattern, either long-term potentiation-like (LTP-like) (intermittent TBS, iTBS) or long-term depression-like (LTD-like) [continuous TBS (cTBS)] effects can be induced in a circuit-specific way (13, 14). A recent study from our laboratory has shown that in cocaine and alcohol users, respectively, mPFC cTBS can lead to a decrease in drug cue reactivity in the mPFC and downstream subcortical targets, including the striatum (15).

However, it is also known that there are individual differences in responses to rTMS treatment, with some individuals responding as expected and others responding less or not at all (16–19). We recently showed that white matter integrity between the mPFC and putamen was one factor that influences individual differences in striatal response to mPFC cTBS (20). In addition, there is some evidence of state-dependent effects, where baseline neural state influences individual differences in response to rTMS (21–23). The objective of the present study was to assess the striatum as a biomarker of treatment response by determining if baseline drug cue reactivity in the striatum influences striatal response to mPFC cTBS. To accomplish this goal, striatal network activity during the cocaine-cue exposure task was extracted using group independent component analysis (ICA) before and after real and sham cTBS, and baseline striatal cue reactivity was related to striatal treatment response.

## Methods

### Participants and Procedures

Twenty-five nontreatment-seeking chronic cocaine users [13 females; mean (SD) age = 42 (9) years] were recruited from the Charleston, SC, metropolitan area using digital and print media (i.e., Craigslist, bus ads) to participate in this single-blind, active sham-controlled crossover study. Following informed consent procedures approved by the Medical University of South Carolina Institutional Review Board, participants completed assessments related to protocol safety, mental status, and drug use to determine study eligibility (see **Supplemental Materials, Methods** for detailed inclusion/exclusion information).

Eligible participants completed two MRI/rTMS visits (each ∼1 h). A multi-panel urine drug screen (Quikvue 6-panel urine drug screen, Quidel, San Diego, CA) was given to ensure participants were not under the influence of cocaine, [meth]amphetamine, opiates, benzodiazepines, and marijuana during study sessions. A breathalyzer was given to ensure that participants were not under the influence of alcohol. All participants received real cTBS (FP1 landmark based on electroencephalogram (EEG) 10–20 system, 110% resting motor threshold) and sham cTBS (order counterbalanced across participants, six 600-pulse sessions of cTBS on each visit, 60-s break after 1,800 pulses). Functional MRI (fMRI) data were collected immediately before and after exposure to cTBS (see **Supplemental Materials Figure S1** for study design). Visit 2 occurred 7 to 14 days after visit 1. The second cue reactivity fMRI scan was initiated within 10 min of receiving cTBS and completed no later than 30 min after cTBS to maximize presumed effects of cTBS on cortical activity (11). Self-reported cocaine craving was assessed upon visit initiation, before the baseline fMRI scan, before the cTBS session, immediately after cTBS, and immediately after the second fMRI scan.

**Clinical Assessments.** Self-report assessments included the Structured Clinical Interview for the Diagnostic and Statistical Manual of Mental Disorders IV (SCID for DSM-IV) (SCID) (24), Timeline Follow-back (TLFB; for cocaine, alcohol, marijuana, and nicotine) (25), Alcohol Use Disorders Identification Test (AUDIT) (26), Fagerstrom Smoking Inventory (27), Beck’s Depression Inventory (BDI-II) (28), and Spielberger State-Trait Anxiety Inventory (STAI) (29). TLFB was used to evaluate past week’s substance use at screening and both MRI/rTMS visits. In addition, a brief cocaine craving assessment (scale 1–10: 1, no craving; 10, high craving) was administered at five time points during both MRI/rTMS visits to monitor craving levels throughout the study (see **Figure S1**). As typically done in cue-induced craving studies, study personnel ensured craving levels were at or below baseline before participants were dismissed from each visit. Participants received monetary compensation for their time and effort and travel to and from the university.

**Cocaine Cue Reactivity fMRI Task.** The cocaine cue reactivity task was administered in the MRI scanner as a block design using E-Prime software (Psychology Software Tools, Inc.; 30). The total task time was approximately 12 min and consisted of six 120-s epochs. Each epoch included alternating 24-s blocks of four task conditions: *Drug*, *Neutral*, *Blur*, and *Rest*, with each block followed by a 6-s cocaine craving inquiry where participants were asked to rate their current cocaine craving level on a 1 to 5 rating scale (1, none and 5, high). The task conditions included images of cocaine-related stimuli (e.g., crack pipe; users snorting cocaine), neutral stimuli (e.g., cooking utensils; people eating dinner), blurred stimuli acting as visual controls by matching cocaine images in color and hue, and a fixation cross for alert rest periods. During each task block, five images were presented (4.8 s). Task blocks were counterbalanced across epochs.

**Neuroimaging.** Participants were scanned using a Siemens 3.0T Tim Trio (Siemens Medical, Erlangen, Germany) MRI scanner with a 32-channel head coil. High-resolution T1-weighted structural images were acquired using a magnetization prepared gradient echo (MPRAGE) sequence (repetition time/echo time = 1,900 ms/2.34 ms; field of view = 220 mm; matrix = 256 × 256 voxels; 192 slices; slice thickness = 1.0 mm with no gap; final resolution = 1 mm^3^ voxels). Functional images were acquired with a multislice gradient-echo echo planar imaging (EPI) sequence (repetition time/echo time = 2,200 ms/35 ms; field of view = 192 mm; matrix = 64 x 64 voxels; 36 slices; slice thickness = 3 mm with no gap; final resolution = 3 mm^3^ voxels). Each functional run consisted of 328 time points.

**cTBS Protocol — Real and Sham cTBS.** Coil position was determined using standardized coordinates from the EEG International 10–20 system (with FP1 corresponding to the left mPFC stimulation target). The location and orientation of each participant’s coil placement was indicated on a nylon cap that participants wore throughout visit 1 and both MRI/rTMS sessions. Participants’ resting motor threshold (rMT; stimulation intensity applied over left motor cortex to produce 50% motor evoked potential response rate in contralateral abductor pollicis brevis) was identified using the standardized PEST procedure (31). The stimulation dose applied to the mPFC was set to be 110% rMT due to the larger scalp-to-cortex distance for PFC versus motor cortex requiring a larger dose to attain equivalent effects (32). The cTBS treatment was administered with a figure-of-eight MagPro Cool-B65 A/P coil (MagVenture, Farum, Denmark). Participants received two 2-min trains of cTBS over FP1 (1 train = 120 s; 3 pulse bursts at 5 Hz; 15 pulses/s; 1,800 pulses/train; 60-s intertrain interval). To enhance tolerability, stimulation intensity was gradually escalated in 5% increments (from 80% to 110% rMT) over the first 30 s of each train.

The Magventure MagPro system includes an integrated active sham. When the coil was oriented in the treatment position, real cTBS was administered, and the scalp electrodes placed on the left frontalis muscle under the coil were not active. When the coil was flipped 180°, the active side of the coil faced *away* from the scalp. In this configuration, the sound and pressure of the coil remained constant and the scalp electrodes became active, thus mimicking the multi-sensory experience of real cTBS, without the CNS stimulation. Previous studies in our laboratory have demonstrated that participants are unable to differentiate real from sham stimulation, with participants exhibiting ∼48% accuracy (i.e., ∼chance) in identifying whether they received real or sham cTBS in a given session (33). However, for continued assurance, participants were surveyed after each session to routinely assess the integrity of the blinded study.

**Cue Recollection During cTBS Administration.** Before cTBS administration, participants were asked to recall the last time they used cocaine, and using a series of standardized questions from traditional Narrative Exposure Therapy practice (34), they were asked to describe the place they were using, a visual description of the scene, and a description of the sensory properties of the drug including taste, smell, and sensation. During cTBS administration, the participants were primed every 20 s to “Think about that scene you described wherein you were last using cocaine/crack” (paraphrased such that this was tailored to the participant’s description).

### Data Analysis

**Neuroimaging Preprocessing.** MRI data were preprocessed using SPM12 (Wellcome Department of Cognitive Neurology, London, UK) implemented in Matlab 7.14 (MathWorks, Inc., Natick, MA; see **Supplemental Materials, Methods** for preprocessing details). Of the 25 recruited participants, 6 participants were excluded for excessive head motion artifact (>3 mm in any plane; x, y, z, roll, pitch, yaw; see **Supplemental Materials, Methods** for details). Data analyses were conducted on the remaining 19 participants [11 males; mean (SD) age = 41 (10) years; range, 21–54 years; see **Table 1** for demographics].

**Table 1:** Descriptive demographic, clinical, and drug use statistics.

	*n* = 19 Total sample	*n* = 10 cue-sensitive	*n* = 9 cue-insensitive	Cue-sensitive vs. cue-insensitive
**Demographics**				
Sex	11 M, 8 F	6 M, 4 F	5 M, 4 F	χ*^2^* 0.04
Age	41.2 (± 9.5) years	42.7 (⺠± 8.7) years	39.4 (⺠± 10.0) years	*T* 0.72
Ethnicity	18 AA, 1 C	9 AA, 1 C	9 AA	χ*^2^* 0.95
Education	12.2 (± 1.4) years	12.2 (± 1.8) years	12.1 (± 0.7) years	*t* 0.13
**Cocaine use**				
Preferred drug administration	10 smoke, 8 snort, 1 both	5 smoke, 4 snort, 1 both	5 smoke, 4 snort	χ*^2^* 0.95
Age of first cocaine use	22.4 (± 5.7) years	20.3 (± 4.3) years	24.7 (± 6.2) years	*t* -1.71
Total duration of cocaine use	18.8 (± 9.4) years	22.4 (± 9.8) years	14.8 (± 7.1) years	*t* 1.82
Amount $ spent per week	$136.71 (± $98.70)	$147.80 (± 110.00)	$124.40 (± 82.70)	*t* 0.49
Days used in last 30 days	11.3 (± 6.9) days	10.5 (± 4.8) days	12.1 (± 8.5) days	*t* -0.49
Time since last use (at visit)	2.4 (± 1.0) days	2.3 (± 1.1) days	2.6 (± 1.0) days	*t* -0.51
**Other substance use**				
Nicotine smokers	17 (89%)	9 (90%)	8 (89%)	χ*^2^* 0.39
Nicotine severity (Fagerström)	3.1 (± 1.9)	2.8 (± 2.1)	3.1 (± 1.9)	*t* -0.32
Marijuana smokers	14 (74%)	7 (70%)	7 (78%)	χ*^2^* 0.15
Days MJ used in last 30 days	4.4 (± 9.0) days	3.4 (± 6.8) days	5.3 (± 10.2) days	*t* -0.37
Alcohol use severity (AUDIT)	9.2 (± 5.3)	10.6 (± 3.8)	7.7 (± 6.2)	*t* 1.18
Age first alcohol use	17.0 (± 3.3) years	17.7 (± 4.5) years	17.1 (± 1.5) years	*t* 0.34
**Mental status**				
Depressive symptoms (BDI)	10.6 (± 9.1)	12.3 (± 10.9)	8.8 (± 6.0)	*t* 0.82
State Anxiety (STAI-S)	37.4 (± 12.3)	34.0 (± 12.2)	41.2 (± 11.2)	*t* -1.26
Trait Anxiety (STAI-T)	40.7 (± 12.2)^?^	41.4 (± 13.4)^?^	39.9 (± 10.8)	*t* 0.26^?^
**Treatment-related measures**				
Scalp-to-cortex distance (mm)^¥^	17.9 (± 3.7) mm	17.3 (± 3.9) mm	18.9 (± 3.1) mm	*t* -0.84
Mean absolute cTBS dose^%^	57% (± 9%)	61% (± 9%)	52% (± 8%)	*t* -0.25
Baseline cocaine craving	3.3 (± 2.0)	3.9 (± 2.3)	2.7 (± 1.6)	*t* 1.30
Change in cocaine craving^¤^	-0.6 (± 1.9)	-0.6 (± 1.3)	-0.5 (± 2.4)	*t* -0.11
Baseline striatum reactivity (⺠β)	0.0 (± 0.3)	0.2 (± 0.2)*	-0.3 (± 0.2)*	*t* 5.96**
Change striatum reactivity (Δβ)	-0.1 (± 0.5)	-0.4 (± 0.4)*	0.5 (± 0.3)*	*t* -5.36**

M, males; F, females; AA, African-American; C, Caucasian; MJ, marijuana; AUDIT, Alcohol Use Disorders Identification Test; BDI, Beck’s Depression Inventory; STAI, Spielberger State-Trait Anxiety Inventory. Values either indicate mean (± standard deviation) or count (percent%). ^ǂ^Missing STAI-T score for one participant. ^¥^Scalp-to-cortex distance (mm) for mPFC coil placement at EEG 10-20 FP1. ^%^Mean absolute dose of cTBS administered (% total machine output to achieve 110% rMT, averaged over both stimulation sessions). ^¤^Change in craving values given by: Δafter real cTBS - Δafter sham. Significance after multiple comparison adjustment (adjusted p < .05*; p < .005**).

**Independent Component Analysis.** To accomplish the primary objective of the present study, which was to assess the impact of baseline striatal network drug cue reactivity on cTBS treatment response, the temporal dynamics of the striatal network as a whole were isolated using group-level ICA. Specifically, group spatial ICA was conducted on all 76 cue reactivity task fMRI data sets [4 per participant (pre/post-real, pre/post-sham) × 19 participants] using Matlab’s Group ICA of fMRI toolbox (GIFT) (35) (see **Supplemental Materials, Methods** for detailed spatial ICA methods). Briefly, the GIFT ICA procedure uses a two-step data reduction approach. In the first step, principal component analysis (PCA) reduced each subject’s data set into 100 subject-specific principal components. For the second step, subject-specific principal components were concatenated and further reduced into 50 group-level principal components, which were then entered into the final group ICA for identification of the 50 group-level independent components. The component reliability was determined by a stability index (20 iterations of ICASSO, Infomax algorithm). Each independent component’s subject-specific representation (i.e., unique spatial map and time course) was computed *via* back-reconstruction of the group independent components. These data were normalized to *z* scores to enable comparison across subjects.

**General Linear Modeling (GLM) of ICA Network Time Courses.** Each subject-specific striatal network time course was entered into a general linear regression [Analysis of Functional Neuroimages’ (AFNI’s) 3dDeconvolve] with five task conditions (*drug, neutral, blur, rate_craving_drug, rate_craving_other*) and six movement parameters as regressors. For each subject, a mean beta weight value (β) was estimated for the striatal network, which provided a single measure of the level of task-related activity for the network as a whole during each of the task conditions (35–38). Striatal network drug cue reactivity was computed by contrasting network activity during drug cue versus neutral cue conditions. Network drug cue reactivity *after* real/sham cTBS was compared with engagement *before* real/sham cTBS using a factorial design and *post hoc* paired *t* tests.

### Linear Regression to Identify Predictors of Neural Response to mPFC cTBS Treatment

***Baseline Striatum Drug Cue Reactivity.*** Robust linear regression was used to determine the association between baseline striatum drug cue reactivity and changes in drug cue reactivity after real (vs. sham) mPFC cTBS. Robust regression was performed in Matlab using iteratively reweighted least squares with a Huber weighting function (default weighting parameter, 1.345). Robust regression was preferred over standard least-squares linear regression due to its minimization of the influence of response variable outliers (39, 40). The regression included baseline striatum network drug cue reactivity as the predictor variable and change in striatum network reactivity after real (vs. sham) cTBS as the outcome variable.

***Clinical, Demographic, and Drug Use History Variables.*** To determine whether clinical and demographic variables influenced or predicted cTBS treatment outcomes, hierarchical multiple linear regressions were conducted with clinical and demographic variables of interest as the predictors and covariate predictors and striatum network reactivity after real (vs. sham) cTBS as the outcome variable.

***Scalp-to-Cortex Distance.*** Given that the effects of TMS on cortical excitability are proportional to the distance between the skull and cortex (32, 41), we calculated the distance from the scalp to cortex on the transverse plane of MPRAGE images for each participant (see **Supplemental Materials, Results**). The average distance from participant-specific placement of FP1 to the cortex was 18 mm (±3.7 mm). These distances were incorporated into the analyses as covariates.

## Results

**Figure 1:**
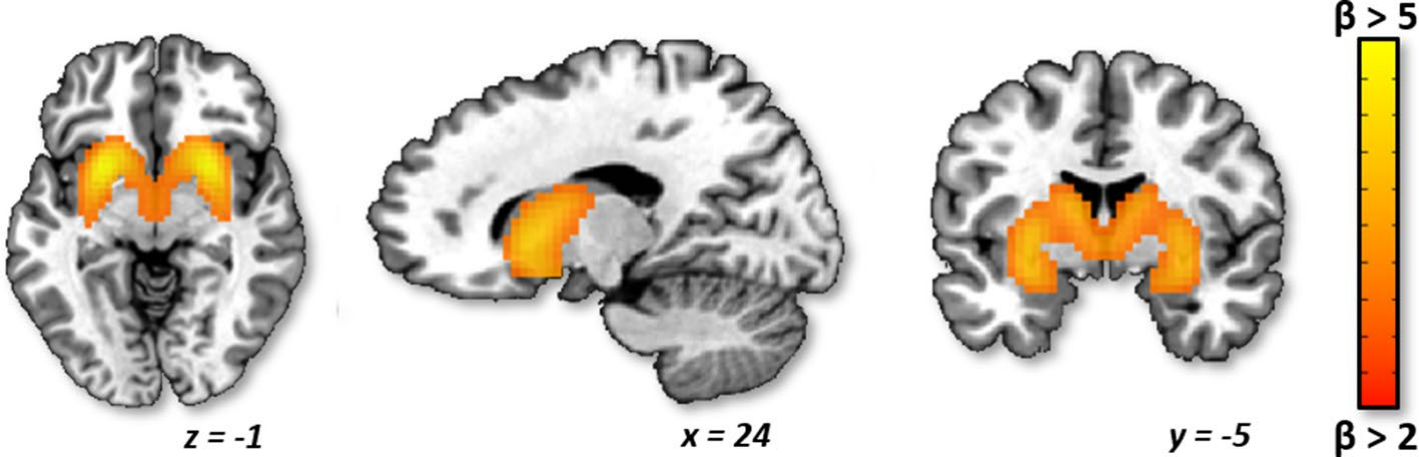
Striatum independent component analysis (ICA) network. Axial, sagittal, and coronal planes are shown, respectively, for the group average striatum network component, which includes the bilateral caudate, putamen, and nucleus accumbens. The network is depicted in neurological convention (left = left) in Montreal Neurological Institute (MNI) coordinate space with cluster-level threshold at β > 2 and minimum cluster size = 50 voxels.

**Identification of Striatum ICA Component.** Of the 50 components identified by ICA, 17 were classified as noise components (i.e., corresponding to motion and/or other signal artifacts). The 33 non-noise components were comprised of several canonical functional networks commonly associated with sensory, motor, cognitive, and affective processing (42, 43). However, given our focus on evaluating striatum craving circuitry, we focused on the striatum network component, which encompassed bilateral caudate, putamen, and nucleus accumbens (**Figure 1**). Evaluation of the back reconstruction of the striatum component onto the 76 participant data sets confirmed that all participant data sets exhibited a robust striatum network component, including a subject-specific striatum spatial map and time course.

### Effect of mPFC cTBS Treatment on Striatum Network Activity During Drug Cue Exposure

**Figure 2:**
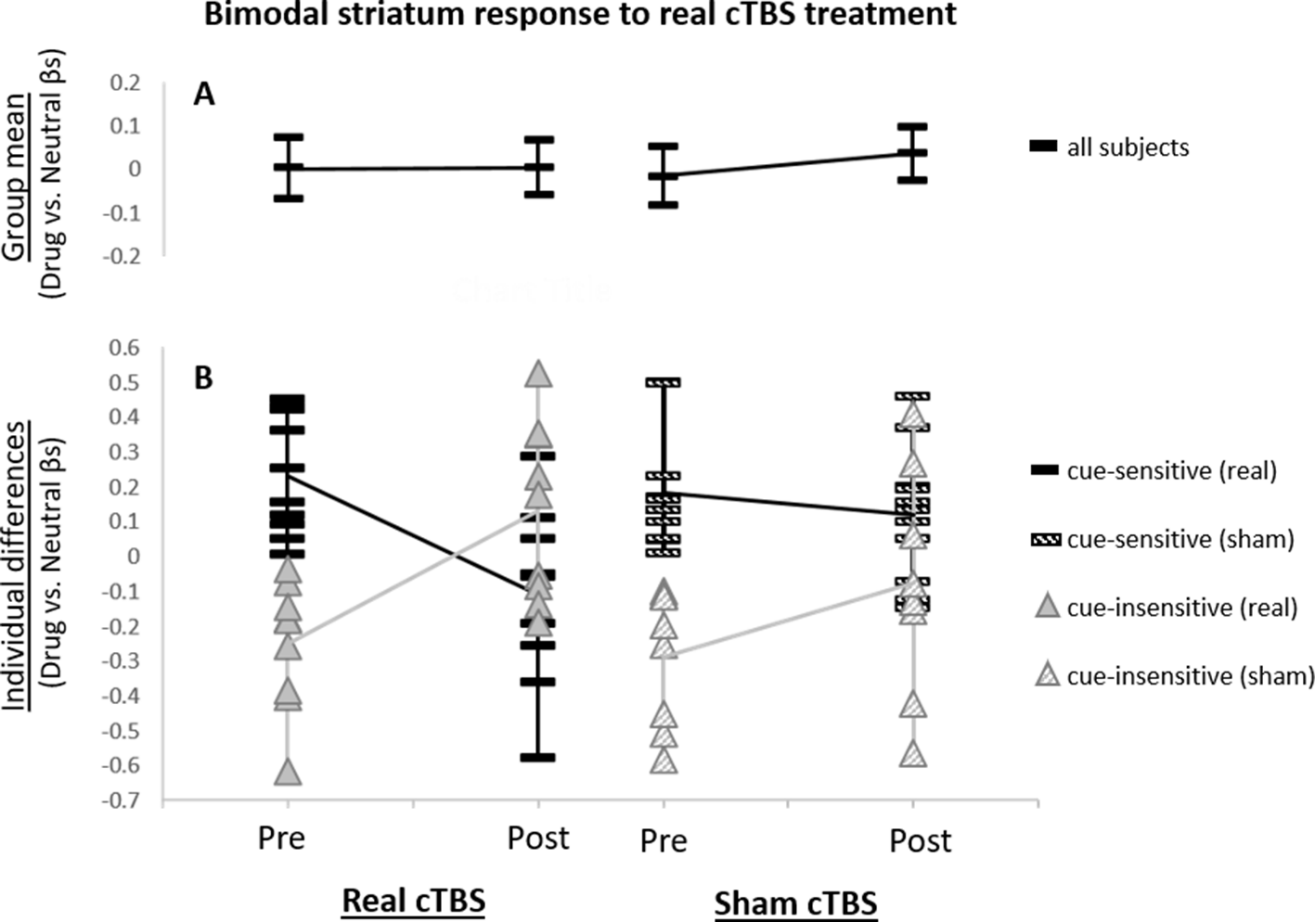
Striatum network drug cue reactivity before and after real and sham continuous theta burst stimulation (cTBS). **(A)** For the group, striatum network did not exhibit significantly elevated drug cue reactivity for any of the functional magnetic resonance imaging (fMRI) scans (*p* > 0.05 for pre- and post-real cTBS and sham, respectively). **(B)** Individual differences analysis revealed a bimodal neural response to real cTBS. Participants, who had initially exhibited elevated striatum network drug cue reactivity (*t*_9_ = 4.34; *p* ≤ 0.005), revealed significantly attenuated activity after real cTBS (*t*_9_ = −3.76; *p* ≤ 0.005; black bars). Subjects, who had initially exhibited suppressed network drug cue reactivity (*t*_8_ = −4.09; *p* ≤ 0.005), revealed significantly enhanced activity after real cTBS (*t*_8_ = 4.01; *p* ≤ 0.005; gray triangles). No significant differences were found for sham (striped bars/triangles).

***Group Analysis.*** Across all subjects, there was no significant elevation of striatal network activity during drug cue exposure at any time point (**Figure 2A**). Additionally, across all subjects, there was no significant attenuation of striatal network cue reactivity following real versus sham cTBS (*F* *_1,68_* = 0.17; *p* > 0.05; **Figure 2A**).

***Individual Differences Analysis.*** Analysis of individual differences, however, revealed that real cTBS *did* strongly alter striatum drug cue reactivity but was modulated by participants’ baseline striatum network cue reactivity (**Figure 2B**). Specifically, “cue-sensitive” participants who were responsive to cue induction and initially exhibited *elevated* drug cue reactivity (*t*_9_ = 4.34; *p* ≤ 0.005), revealed significantly *attenuated* activity after real (vs. sham) cTBS (*t*_9_ = -3.76; *p* ≤ 0.005; **Figure 2B**, *black bars*). “Cue-insensitive” participants, who were not responsive to cue induction and initially exhibited *suppressed* drug cue reactivity (*t*_8_ = -4.09; *p* ≤ 0.005), revealed significantly *enhanced* activity after real (vs. sham) cTBS (*t*_8_ = 4.01; *p* ≤ 0.005; **Figure 2B**, *gray triangles*). These strongly opposing neural responses canceled each other out in the group-level analysis, at both time points, thus causing the group-level analyses to appear non-significant. Conversely, no statistically distinct response patterns were identified for sham stimulation (**Figure 2B**; *striped bars/triangles*). Thus, these data convey a bimodal neural response profile for real (vs. sham) mPFC cTBS (paired *t* test for cue-sensitive vs. cue-insensitive subjects: *t*_17_ = -5.36; *p* < 0.005) and an overall significant three-way interaction between treatment type (real/sham), time (pre/post), and baseline cue reactivity (cue-sensitive/cue-insensitive) (*F* *_1,68_* = 11.83, *p* = 0.001). These results are not likely to reflect regression to the mean, as this bimodal response pattern was only seen for the real cTBS condition and not for sham, whereas in the case of regression to the mean, this pattern would be expected for both conditions. In addition, in a *post hoc* analysis, we assessed whether treatment order (real or sham first in this crossover design) influenced treatment response and found no effect.

**Figure 3:**
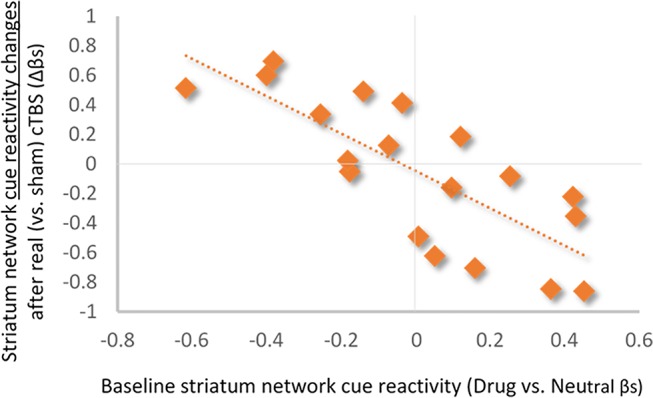
Relationship between baseline striatum network drug cue reactivity and change in drug cue reactivity after real (vs. sham) cTBS treatment. Baseline striatum drug cue reactivity was strongly inversely related to striatum network response to real (vs. sham) cTBS (ρ = −0.79; *p* < 0.001; *R* ^2^_Adj_ = 0.58).

**Baseline Striatum Drug Cue Reactivity Predicts Changes in Striatum Network Response to Real Versus Sham mPFC cTBS.** Baseline striatum network drug cue reactivity was strongly inversely related to striatum network reactivity following real (vs. sham) mPFC cTBS (⺠ρ = -0.79; *p* < 0.001; *R* ^2^_Adj_ = 0.58; **Figure 3**).

**Figure 4:**
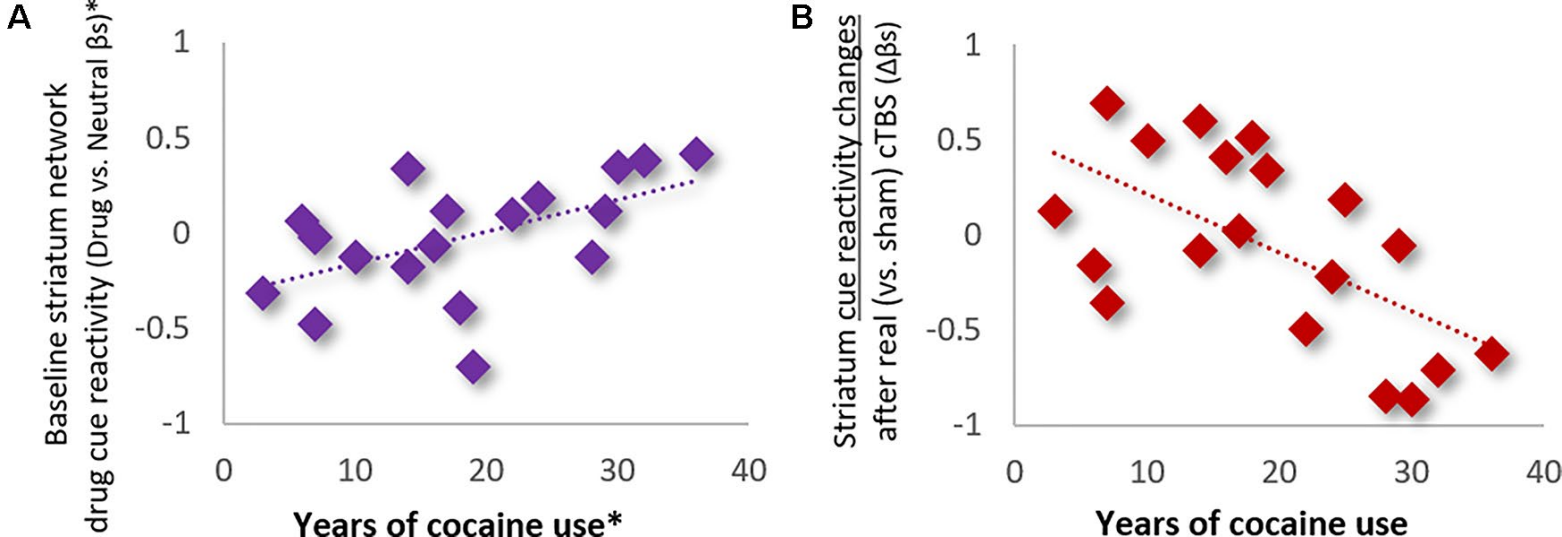
Years of cocaine use was **(A)** positively related to baseline striatum network reactivity to drug cues (ρ = 0.67; *p* < 0.01; *R* ^2^_Adj_ = 0.45; *Controlling for route of drug administration and nicotine dependence), and **(B)** inversely related to network response to real (vs. sham) cTBS treatment (ρ = −0.57; *p* < 0.01; *R* ^2^_Adj_ = 0.32).

***Influence of Clinical, Demographic, and Drug Use History Variables.*** Analysis of the influence of clinical and demographic variables on treatment outcomes (see **Table 1** for variables assessed) revealed that only the total years of cocaine use was a significant modulator of striatum drug cue reactivity—for both baseline and treatment-related changes (**Figure 4**). Specifically, hierarchical multiple linear regression showed that the years of cocaine use was strongly positively related to baseline striatum cue reactivity (ρ = 0.67; *p* < 0.01; *R* ^2^_Adj_ = 0.45; when controlling for route of drug administration and Fagerström nicotine dependence; **Figure 4A**) and strongly inversely related to changes in striatum cue reactivity after real (vs. sham) cTBS treatment (ρ = -0.57; *p* < 0.01; *R* ^2^_Adj_ = 0.32; **Figure 4B**). However, despite strong correlations between baseline striatum cue reactivity and years of cocaine use, these variables each explained unique variance in striatum network response to real (vs. sham) cTBS treatment.

## Discussion

In our previous study, we showed that mPFC cTBS could attenuate drug cue reactivity in both the mPFC and striatum in cocaine and alcohol users (15, 20). The present study extends these data by demonstrating that individual variability in the effect of mPFC cTBS on striatal circuitry may be related to baseline striatal reactivity to cues. Thus, our preliminary findings in this small data set are a first glimpse into informing treatment by suggesting that cocaine users with the greatest striatum network reactivity to drug cues at baseline may “benefit” most from mPFC cTBS treatment and that individuals with low baseline striatal reactivity to drug cues may not make good treatment candidates.

**Individual Variability in Response to Theta Burst Stimulation.** Although, to date, there have only been a handful of therapeutic neurostimulation studies implementing mPFC-targeted rTMS [see Ref. (13) for review], individual variability observed in the present study is consistent with studies of dorsal mPFC (dmPFC) rTMS in major depression (17), eating disorders (18), and obsessive-compulsive disorder (19, 44). Using resting-state fMRI, Dunlop et al. (18) demonstrated that in patients with eating disorders, baseline fronto-striatal connectivity discriminated treatment responders from non-responders, with divergent treatment-related alterations in connectivity corresponding to either symptom improvement or worsening, respectively. This divergence has also been observed when rTMS is applied to other cortical targets (16, 17, 19, 45–51). Together, these studies demonstrate that 1) the effects of various rTMS interventions, especially TBS, can be highly variable within a patient population, but 2) baseline levels of neural activity may be useful biomarkers of an individual’s predisposition to TMS-induced neuroplastic changes.

Individual variability in neural responsiveness to TBS may be related to differences in plasticity potential (aka metaplasticity) of a given neural circuit across individuals. This concept is often referred to as homeostatic metaplasticity, whereby changes in cortical excitability induced by rTMS depend on the history of neural activation (22, 52). Specifically, the Bienenstock–Cooper–Munro (BCM) theory of homeostatic plasticity (53) posits that a history of lower post-synaptic activity will lower the synaptic modification threshold for LTP and increase the threshold for LTD. Conversely, a history of high synaptic activity will shift the modification threshold toward favoring the induction of LTD and increase the threshold for LTP (54).

Therefore, metaplasticity—or the propensity of a neural circuit to experience a plastic change—may be related to the current state of that circuit’s engagement. Several studies involving both animals and humans have provided strong evidence for this phenomenon by showing that the effects of brain stimulation are influenced by prior activation of a given circuit, whether through priming stimulation or physiologic activity (see 21–23 for reviews). Therefore, it is possible that the bimodal neural responses shown across many rTMS studies—where patients with higher baseline neural activity or connectivity show subsequent attenuation, and patients with lower baseline activity or connectivity show subsequent elevation (or facilitation)—are, in fact, evidence of system- or network-level homeostatic metaplasticity (21, 22).

This theory warrants further investigation in human brain stimulation studies (22, 23), particularly in substance abuse populations where other biologic and drug-related factors impact neuroplasticity (5, 13, 20). However, if metaplastic mechanisms do play a significant role in the direction and magnitude of neural response to brain stimulation, then not accounting for or understanding these phenomena may continue to lead to broad variation in rTMS study outcomes (21, 55). It is, therefore, clear that researchers implementing TBS as an intervention in psychiatry should exercise caution in interpreting their study outcomes without considering the role of individual differences in correlates and predictors of response to stimulation. Understanding individual variability and potential mechanisms of metaplasticity in the relevant neural circuits will enable us to optimize the efficacy of rTMS, and TBS in particular, as a treatment tool (22, 23). Therefore, considerations for future implementation of TBS research should involve a focus on identifying the neural, behavioral, and clinical markers that predict clinically relevant outcomes to treatment.

**The Utility of fMRI as a Biomarker.** In particular, studies like the present, which use functional neuroimaging to inform brain stimulation, are of critical importance to characterizing and developing therapeutic neuromodulation techniques (13, 17, 18). Specifically, the present fMRI task data revealed the neural predictors and correlates of mPFC cTBS response and provided support for homeostatic metaplasticity as a potential neural mechanism for divergent treatment outcomes. Thus, fMRI was of both clinical and neuroscientific relevance, indicating potential treatment candidacy while also illuminating avenues for investigating neuromodulatory mechanisms.

Given that the primary goal of this study was to assess the striatum (the primary projection of mPFC neurons) as a biomarker for treatment response to mPFC-targeted cTBS, we utilized a data-driven ICA to capture changes in the temporal dynamics of striatal network task engagement. ICA was used in the present study versus traditional univariate or Region-of-interest (ROI)-based methods for three primary reasons: 1) the data-driven basis of ICA enabled extraction of the intrinsic spatiotemporal structure of the striatum network in this population without relying on *a priori* input (35, 38, 56, 57); 2) ICA’s multivariate statistical approach permitted the measurement of the engagement of the striatum network as a whole, such that the multifocal brain areas simultaneously activated during the cue reactivity task could be captured in their overall patterns of association, rather than being assessed voxelwise or as ROI pairs (38, 57, 58); and 3) increased sensitivity in detecting task-related changes in fMRI signal would result from ICA’s ability to diminish noise in the final output by separating artifact from real fMRI signal (36, 59–61). As such, ICA was selected for identification and characterization of the striatum network to enable measurement of network-level task engagement in the subsequent task analysis. However, although focusing on the striatum network was appropriate to address our primary research question, it did not enable us to make conclusions about other brain regions, which may also be affected by the task and mPFC cTBS treatment protocol. As such, these questions could be addressed through further investigation of other relevant cognitive and affective networks, identified through ICA or through a whole-brain, general linear model approach. Although this was beyond the scope of the present research investigation, it would be a valuable approach for future investigation.

The primary limitation of the present study is that it only involves 1 day of cTBS treatment. Although the participants received six 600-pulse sessions of cTBS on that day, there is conflicting evidence as for whether a single day of brain stimulation is sufficient to induce sustainable neural changes (11, 12, 62, 63). Relatedly, we recently showed, in a subset of these subjects, that a single session of mPFC cTBS produced neural changes, but did not produce changes in drug cue-induced craving (15). However, it is generally recognized that a single day of rTMS is likely not sufficient to produce changes in complex behaviors, such as craving, because rTMS effects are cumulative, and it often takes multiple sessions of treatment for clinically meaningful responses in behavior to emerge (64–67). These data are, however, an important “proof of principle,” demonstrating that it is not only possible to shift neural reactivity to cocaine cues in a single day using rTMS but also that individual differences in neural response to rTMS are state dependent, which is an important, foundational step toward determining the efficacy of mPFC cTBS as a treatment for substance abuse. Additionally, the sample size is relatively small compared with many clinical treatment studies in cocaine users. However, it is similar in size to many currently published rTMS studies in cocaine dependence (33, 68)—none of which have used neuroimaging as a predictor of response.

These preliminary findings provide the first demonstration that striatal network activity patterns during drug cue exposure fMRI tasks may be sensitive predictors of response to rTMS treatment and can be used to refine treatment selection and monitor outcomes. However, variability in neural response to treatment and lack of significant changes in cocaine craving indicate the need to further study the neurobiological and technical parameters of successful therapeutic stimulation in substance abuse.

## Data Availability Statement

The datasets generated for this study are available on request to the corresponding author.

## Ethics Statement

All subjects gave oral and written informed consent in accordance with the Declaration of Helsinki. The protocol was approved by the Medical University of South Carolina Institutional Review Board.

## Author Contributions

CH was responsible for the concept and design of the overall research study. OM and WD were responsible for study administration and data collection. LD preprocessed MRI data. TK-R designed and executed data analyses, interpreted results, and drafted the manuscript. CH and MG provided critical revision of the manuscript for intellectual content. All authors critically reviewed and approved the final version for publication.

## Funding

This research was supported by the National Institutes of Health grants R01DA036617 (CH), P50DA015369 (Kalivas), P50AA010761 (Becker), and T32DA007288 (McGinty). Additional support was provided by the South Carolina Translational Research Institute UL1TR000062 and R25DA033680.

## Conflict of Interest Statement

The authors declare that the research was conducted in the absence of any commercial or financial relationships that could be construed as a potential conflict of interest.
