# Supplementary material for: State-Dependent Effects of Ventromedial Prefrontal Cortex Continuous Thetaburst Stimulation on Cocaine Cue Reactivity in Chronic Cocaine Users
Source: Front Psychiatry. 2019 May 8;10:317. doi: 10.3389/fpsyt.2019.00317 (PMC6517551; doi:10.3389/fpsyt.2019.00317)
Supplement: Supplementary file 1 [file DataSheet_1.docx]

1. **SUPPLEMENTAL MATERIALS**

**SUPPLEMENTAL METHODS**

**Study design.** Fig. S1 illustrates the timeline for each of two MRI/rTMS visits.


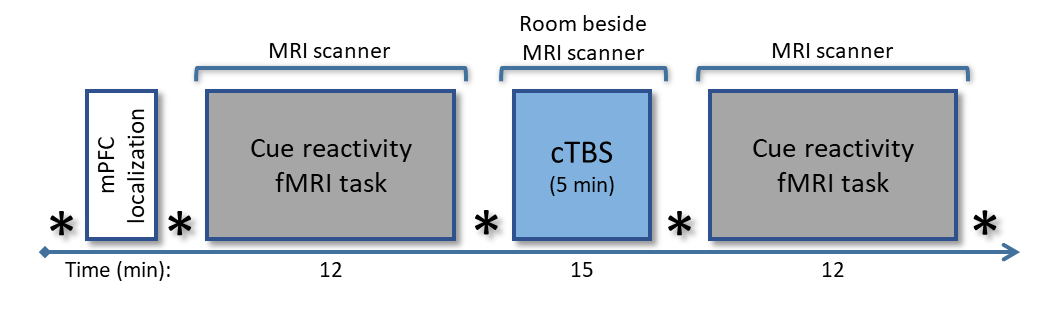


**Figure S1. MRI/rTMS Visit.** The cocaine cue reactivity fMRI task was administered before and after mPFC cTBS in non-treatment-seeking chronic cocaine users. In this active sham-controlled crossover design, participants were randomized to receive real or sham cTBS counterbalanced across two MRI/rTMS visits. Self-reported cocaine craving was reported at five time points (*) throughout the study.

**Inclusion/Exclusion Criteria.** All individuals were screened with the Structured Clinical Interview for DSM-IV Axis I Disorders (SCID; Ventura et al., 1998). Inclusion criteria required that participants meet DSM-IV criteria for Cocaine Dependence; be aged 21-55 years; not currently taking any psychoactive medications; and have a negative medical history for seizures. Exclusion criteria included current use of prescription or illicit psychoactive drugs other than cocaine and marijuana; DSM-IV current or past substance dependence criteria other than cocaine; smoking >1 pack of cigarettes per day; current breath alcohol concentration >0.002; a lifetime history of head injury; a history of seizures or migraine headaches (as TMS may exacerbate these conditions); ferromagnetic implants and other contraindications to the high-field MRI environment (e.g. claustrophobia); and pregnancy (determined through urinalysis).

**Neuroimaging Preprocessing.** MRI data were preprocessed using SPM12 (Wellcome Department of Cognitive Neurology, London, UK) implemented in Matlab 7.14 (MathWorks, Inc., Natick, MA). MR Images were first converted from DICOM format to 4D NIfTI files, and motion corrected (Realign: 6 parameter rigid-body realignment to first image in each timeseries using a least squares approach). Normalization parameters, bias correction and anatomical tissue maps were determined simultaneously, using the Segment toolbox. Individual anatomical images were stripped of their skulls by masking the bias corrected image with the combined tissue masks of grey matter, white matter and CSF. The functional images derived from realignment were coregistered, through the mean image, to the skull stripped anatomical image (Coregister: Estimate, using normalized mutual information). Coregistered images were then normalized (Normalize: Write) to MNI template space with the nonlinear warps derived from the Segment tool. Finally, functional images were masked (to remove the skull) and smoothed (8mm FWHM Gaussian kernel) to facilitate subsequent between-subject analysis. After stringent artifact removal in the 25 recruited participants (see Tohka et al., 2008 for details on artifact removal process), 6 participants were excluded due to excessive motion artifact (>3mm in any plane; X, Y, Z, roll, pitch, yaw) persisting after preprocessing, thus resulting in a final sample of 19 participants.

**Independent Component Analysis (ICA) Procedure.** Group spatial ICA was conducted on all 19 participants’ cue reactivity task datasets using Matlab’s Group ICA of fMRI Toolbox (GIFT v1.3; Calhoun et al., 2001). Spatial ICA is a data-driven, multivariate statistical approach that can be used to identify networks of neural activation without *a priori* input by separating fMRI data into spatially independent groups of temporally-correlated brain regions, or functional networks (McKeown et al., 1998; Calhoun et al., 2001). The number of independent components for which to solve was determined through (1) GIFT’s minimum description length (or MDL) algorithm determination of optimal model order followed by (2) permutation testing, which involved the evaluation of component quality across several different ICA procedures. ICA was conducted solving for 30, 40, 50, and 60 independent components in order to find the model with, both, the best component stability (given by the stability index, or “iq”) and the best replication of well-supported (“canonical”) functional networks (Ray et al., 2013; Smith et al., 2009). Of the 4 models, solving for 50 group ICA components produced the best balance between component stability (iq ≥ .90) and the partitioning of components into well-validated canonical functional networks.

The GIFT ICA procedure uses a two-step data reduction approach. In the first step, principal component analysis (PCA) reduced each subject's dataset into 100 subject-specific principal components (with the following parameter settings, as detailed in the GIFT v1.3 User Manual: expectation maximization; stacked data sets; full storage of covariance matrix to double precision; and selective eigenvariate solvers). For the second step, subject-specific principal components were concatenated and further reduced into 50 group-level principal components, which were then entered into the final group ICA for identification of the 50 group-level independent components. The ICASSO toolbox was used to determine the reliability of the ICA components; ICASSO was repeated 20 times using the Infomax algorithm to calculate the stability index (iq) of each component, a measure of how reliably each component was reproduced in the sample across iterations. A back-reconstruction was performed using the GICA3 algorithm to identify each independent component’s subject-specific representation (i.e. unique spatial map and timecourse). Finally, all data were normalized to *z*-scores to enable comparison across subjects.

**SUPPLEMENTAL RESULTS**

**Influence of Scalp-to-cortex Distance on the Association Between Baseline Striatum Cue Reactivity and cTBS Treatment Response.** We calculated the distance from the scalp to cortex on the transverse plane of MPRAGE images for each participant. The average distance from the participant-specific placement of FP1 to the cortex was 18mm (± 3.7mm). These distances were incorporated into the primary regression analysis as covariates. We found that when including STC distance as a covariate in the regression between baseline striatum drug cue reactivity and treatment-related changes in cue reactivity, STC distance did not significantly relate to treatment response (β = -0.14, *p* > .05), nor did it significantly modulate the association between baseline cue reactivity and changes in cue reactivity, which remained highly significant (= .44, β = -1.28, *p* < .005).


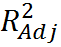

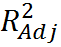


**REFERENCES**

Calhoun, V.D., Adali, T., Pearlson, G.D. and Pekar, J.J. (2001), A method for making group inferences from functional MRI data using independent component analysis. Hum. Brain Mapp., 14: 140–151. doi: 10.1002/hbm.1048.

McKeown, M.J., Jung, T.P., Makeig, S., Brown, G., Kindermann, S. S., Lee, T.W., & Sejnowski, T.J. (1998). Spatially independent activity patterns in functional MRI data during the stroop color-naming task. Proceedings of the National Academy of Sciences of the United States of America, 95(3), 803–810.

Ray, K.L., McKay, D.R., Fox, P.M., Riedel, M.C., Uecker, A.M., Beckmann, C.F., Smith, S.M., Fox, P.T., Laird, A.R. (2013). ICA model order selection of task co-activation networks. Front Neurosci, 7, 237. doi: 10.3389/fnins.2013.00237.

Smith, S.M., Fox, P.T., Miller, K.L., Glahn, D.C., Fox, P.M., Mackay, C.E., et al. (2009). Correspondence of the brain’s functional architecture during activation and rest. Proceedings of the National Academy of Sciences of the United States of America. 106(31), 13040–13045. http://doi.org/10.1073/pnas.0905267106.

Tohka, J., Foerde, K., Aron, A.R., Tom, S.M., Toga, A.W., & Poldrack, R.A. (2008). Automatic independent component labeling for artifact removal in fMRI. Neuroimage, 39(3), 1227–1245. doi:10.1016/j.neuroimage.2007.10.013.

Ventura, J., Liberman, R.P., Green, M.F., Shaner, A., Mintz, J. (1998). Training and quality assurance with the Structured Clinical Interview for DSM-IV (SCID-I/P). Psychiatry Res, 79, pp. 163–173.
